# A Report Containing a Synoptical View of the Principles of Homæopathy, Contrasted with Those of Medicine

**Published:** 1846-06-01

**Authors:** H. Hoyt

**Affiliations:** Syracuse, N. Y.


*A report containing a synoptical view of the principles of Homœopathy, contrasted with those of Medicine, by* H. Hoyt, M. D., *Syracuse, N. Y.*— The Medical Society of the County of Onondaga, at its semi-annual meeting in February last, adopted a resolution appointing a committee, of which Dr. Hoyt was Chairman, to report on the subject of Homœopathy. In accordance with that resolution, Dr. Hoyt has prepared a report, which has been published by Stoddard & Babcock of Syracuse, making a pamphlet of 70 duodecimo pages. Dr. H. discusses the most prominent fundamental positions assumed by Hahnemann and his ardent disciples. In illustrating their inconsistency and gratuitous assumptions, he finds it impossible to abstain entirely from comments which are calculated to throw ridicule upon the subject. Under most circumstances this would be an objectionable feature in such a paper, but we cannot subject the discussion of Homœopathy to the ordinary rules of criticism. Its absurdity is so glaring, the ridiculous extravagances so palpable to the medical inquirer, that it is difficult to elevate the subject sufficiently to regard it with grave contempt. It is beneath the dignity of deliberate sarcasm, still more below calm philosophical disputation. Hence the medical writer who may undertake to treat of it ever so seriously, is unable to go on with the discussion and preserve a serious demeanor. It was said that two Roman Augurs could not look each other in the face without laughing. How must two Homœopathists regard each other when they meet in consultation! We would give something to witness such an exhibition! Our ancestor doctors used to be accused of conferring together by holding for a respectable number of minutes their golden headed canes to their noses: two Homœopathists in consultation, upon a similar rule of symbols, would be likely to go through the manœuvre which has of late been vulgarly significant of successful trickery boastful of its success, consisting of a thumb resting on the extremity of the nose, the phalanges of the same hand extended, and performing a series of flexor and extensor movements!

Still, ridiculously absurd and supremely contemptible as is the so-called science of Homœopathy, there are not wanting converts from the ranks of the medical profession. A large proportion of these are dishonest fellows, who knavishly pander to human credulity for the sake of gain. Such miserable pretenders would never succeed by honestly practicing their profession, and they are accordingly ready for any new thing of which they can avail themselves for their immediate advantage. But there are others whose defection is, we do them the justice to believe, honest and sincere. What does this prove? It may prove simply that medical men are not exempt from the common liability to become infected with popular delusions; or, it may prove that the practice of medicine in *their* hands has been, in reality worse than no practice at all. We do not doubt that some practitioners, if they candidly compare the results of their practice as Allopathists and Homœopathists, justly arrive at the conclusion that they are more successful in the latter capacity. This, however, is far from showing that Homœopathy is preferable to Allopathy, (we use the latter term in the Homœopathic sense) but it only evinces that they are but poor practitioners,—in other words, patients do better without, than with their practice. It would, indeed, be an unfortunate thing if medicine were to be held responsible for its practical application in the hands of every individual member of the medical profession. It is to be judged by its capabilities in judicious, competent hands, not as it is exemplified by those who make it instrumental of more harm than good. This distinction is not apt to be considered by the public, and still less can it be expected that it will be self-applied by those whose experience would furnish striking instances of the class of practitioners last referred to.

We have, on another occasion, expressed the opinion that any formal efforts by medical men to enlighten those prone to this, or other follies of the same sort, are apt to be worse than useless. The truth is, people who have imbibed a delusion will not be reasoned out of it. Who ever succeeded in reasoning a Millerite out of his infatuation? It is not a matter of ratiocination with those who embrace it: they do not fall in with it because they have given it an investigation, and their understandings have become convinced, but because they are predetermined to believe it. It meets a response in their love of the marvelous—their proneness to be duped. Evidence of its truth is not necessary for them to adopt it, how then is it to be supposed that the mind will appreciate evidence against it! The best way is to let the folly expend itself — give it full sweep; and the less it is opposed, the sooner it will die a natural death, and be buried in the tomb of the Capulets.

With these views, it is no discredit to Dr. Hoyt's pamphlet to say, that we doubt if it make a re-convert. It will be read chiefly by medical men, and those who are sufficiently secure against the infection of the new epidemic. This opinion, indeed, accords very nearly with that of the writer. We quote the concluding paragraph of the preface.

"Hahneman, in his organon makes the following statements, to wit: that persons ignorant of the medical profession were the first to discover the Homœopathic method of cure was the safest, the most *rational* and the least subject to failure. It would afford us satisfaction, if we could in justice, pay to this class of our fellow-citizens, as high a compliment by saying they are our first people, who have found out, that in this respect they are laboring under a gross error. For a people possessed of no rational ideas of the science of medicine, to find out, without study, the *most rational method* of cure, is, it must be confessed by all, who have the least particle of knowledge of the art, a phenomenon, of a strange, if not the strangest character; however, as it is so in law and politics, we suppose it must be so in medicine; and for this reason we are not vain enough to believe, that our efforts at convincing this class of poeple of their folly, will be successful. Nevertheless, we do feel a sort of half-way assurance, that with another class of our fellow citizens, and with our medical friends in particular, the arguments we have used in this report, will be by them, candidly weighed, and duly appreciated. If they turn one aberrant thonght, or individual, to an upright course, our object is more than gained."

